# Computational approaches for Cancer 2017 workshop overview

**DOI:** 10.1186/s12859-018-2502-x

**Published:** 2018-12-21

**Authors:** Sunita Chandrasekaran, Eric Stahlberg

**Affiliations:** 1 Department of Computer and Information Sciences, University of Delaware, Newark, DE USA; 2 Biomedical Informatics and Data Science Directorate, Frederick National Laboratory, Frederick, MD USA

## Overview

As the drive towards precision medicine has accelerated, enabled by an ever-increasing abundance of new data and information, the opportunities and challenges in applying computational approaches in cancer research and clinical applications are also growing dramatically. The onset of exascale computing together with the rapid rise of deep learning as an enabling technology and its potential are reshaping the way computation is being applied across scales of computing, across time and across spatial scales. With legislation in the form of the Twenty-first Century Cures Act as well as efforts of the Beau Biden Cancer Moonshot, these underscore the importance of workshops that bring together interdisciplinary experts and insights across the spectrum of computational approaches for cancer.

Several areas are highlighted as opportunities for innovation as disciplines converge in the interest of accelerating and extending the impact of computational approaches for cancer. As outlined in the 2016 Frontiers of Predictive Oncology and Computing meeting report, four distinct yet strongly inter-related areas of innovation include:

- Patient-centric – opportunities where the patient is closely involved
- Organizational – opportunities for organizations and different interests to collaborate
- Data – opportunities around data and information, involving data acquisition, access, aggregation, sharing and management
- Technology – opportunities emanating from the rapid expansion of new technologies to acquire, transport, store, integrate, analyze and simulate complex biological systems

The 2016 meeting report also identifies conducting workshops as an important next step to bring the communities together, to realize these opportunities for innovative advances.

This supplement in BMC bioinformatics provides the reader a glimpse into areas where current innovations and insights are emerging as efforts in interdisciplinary computational cancer science increase and grow. Developed from the contributions at the Third Computational Approaches for Cancer workshop held at the SC17 conference, this supplement brings together the computational community exploring and using high-performance computing (HPC), analytics, predictive modeling, and large datasets in cancer research and clinical applications. The manuscripts are interdisciplinary, with the common interest in cancer and computation the unifying theme. As such, the manuscripts provide rich opportunities for attendees to learn about future directions, current applications and challenges and build collaborations. Maintaining a perspective of accelerating scientific insights and translation of insights to clinical application for improved patient outcomes, the supplement brings together many interests from across the technology, cancer research and clinical domains.

The authors and submissions listed below contributing to this supplement provide key examples of the use of computational methods across a wide range of cancer research applications, ranging from scalable approaches to explore the molecular domain to enabling scalable population level cancer research and clinical. From a technical perspective, the supplement also provides examples across a wide range of computational technologies, ranging from the use of field-programmable gate arrays and GPUs to designs of throughput systems preparing to take advantage of emerging exascale computing capabilities. And, of course, the supplement highlights applications with emerging capabilities in scalable deep learning while exploring working to address the inherent uncertainty in the classification, characterization, and predictive analytics for complex biological systems epitomized by cancer.

### 2017 Computational approaches for Cancer workshop supplement

Nick Hengartner, Leticia Cuellar, John Hogden, Hong-Jun Yoon, John Qui, Georgia Tourassi and Tanmoy Bhattacahrya.

**CAT: Computer Aided Triage: Improving upon the Bayes risk through ε-refusal triage rules.**

Will Fischer, Sanketh S. Moudgalya, Judith D. Cohn and Garrett Kenyon.

**Sparse Coding of Pathology Slides.**

Ahmed Sanaullah, Martin Herbordt, Chen Yang, Yuri Alexeev and Kazutomo Yoshii.

**Real-Time Data Analysis for Medical Diagnosis Using FPGA-Accelerated Neural Networks.**

Shantenu Jha, Matteo Turilli, David Wright, Jumana Daarsekka, Vivekanandan Balasubramanian, Peter Coveney, Shunzhou Wan and Stefan Zasada.

**High-throughput Binding Affinity Calculations at Extreme Scales.**

Debsindhu Bhowmik, Shang Gao, Michael Young and Arvind Ramanathan.

**Deep Clustering of Protein Folding Simulations.**

Justin Wozniak, Rajeev Jain, Prasanna Balaprakash, Jonathan Ozik, Nicholson Collier, John Bauer, Fangfang Xia, Thomas Brettin, Rick Stevens, Jamaludin Mohl-Yusof, Cristina Cardona, Brian Van Essen and Matthew Baughman.

**CANDLE/Supervisor: A Workflow Framework for Machine Learning Applied to Cancer Research.**

Fangfang Xia, Maulik Shukla, Thomas Brettin, Cristina Garcia-Cardona, Judith Cohn, Jonathan Allen, Sergei Maslov, Yvonne Evrard, Susan Holbeck, James Doroshow, Eric Stahlberg and Rick Stevens.

**Predicting Tumor Cell Line Response to Drug Pairs with Deep Learning.**

Jonathan Ozik, Nicholson Collier, Justin Wozniak, Charles Macal, Chase Cockrell, Samuel Friedman, Ahmadreza Ghaffarizadeh, Randy Heiland, Gary An and Paul Macklin.

**High-throughput Cancer Hypothesis Testing with an Integrated PhysiCell-EMEWS Workflow.**

Hong-Jun Yoon, John Qiu, Thomas Watson, Arvind Ramanathan and Georgia Tourassi.

**Scalable Deep Text Comprehension for Cancer Surveillance on High-Performance Computing.**

Reflecting on the noted areas of opportunity in working with patients, with organizations, with data and with technology, this supplement provides examples of how interdisciplinary communities have successfully converged to explore innovative approaches spanning the range of opportunities. The strong coupling among these areas is also evident, where data and technology are utilized across organizations in the interest of accelerating patient impact for cancer. These examples, while distinct, collectively help lay the foundations and insights needed for critical areas of future integrated cancer learning systems.

