# Correction to: Computational approaches for cancer 2017 workshop overview

**DOI:** 10.1186/s12859-019-2631-x

**Published:** 2019-01-22

**Authors:** Sunita Chandrasekaran, Eric Stahlberg

**Affiliations:** 1 Department of Computer and Information Sciences, University of Delaware, Newark, DE USA; 2 Biomedical Informatics and Data Science Directorate, Frederick National Laboratory, Frederick, MD USA

**Correction to: BMC Bioinformatics 2018, 19 (Suppl 18): 487.**

**https://doi.org/10.1186/s12859-018-2502-x**

It was highlighted that the original article [1] contained errors.

The list of authors and submissions for the supplement contained some outdated information and could be improved for readability and clarity. Below is the reformatted and corrected list.

**CAT: computer aided triage improving upon the Bayes risk through ε-refusal triage rules**

Nicolas Hengartner, Leticia Cuellar, Xiao-Cheng Wu, Georgia Tourassi, John Qiu, Blair Christian and Tanmoy Bhattacharya.

**Sparse coding of pathology slides compared to transfer learning with deep neural networks**

Will Fischer, Sanketh S. Moudgalya, Judith D. Cohn, Nga T. T. Nguyen and Garrett T. Kenyon.

**Real-time data analysis for medical diagnosis using FPGA-accelerated neural networks**

Ahmed Sanaullah, Chen Yang, Yuri Alexeev, Kazutomo Yoshii and Martin C. Herbordt.

**High-throughput binding affinity calculations at extreme scales**

Jumana Dakka, Matteo Turilli, David W. Wright, Stefan J. Zasada, Vivek Balasubramanian, Shunzhou Wan, Peter V. Coveney and Shantenu Jha.

**Deep clustering of protein folding simulations**

Debsindhu Bhowmik, Shang Gao, Michael T. Young and Arvind Ramanathan.

**CANDLE/Supervisor: a workflow framework for machine learning applied to cancer research**

Justin M. Wozniak, Rajeev Jain, Prasanna Balaprakash, Jonathan Ozik, Nicholson T. Collier, John Bauer, Fangfang Xia, Thomas Brettin, Rick Stevens, Jamaludin Mohd-Yusof, Cristina Garcia Cardona, Brian Van Essen and Matthew Baughman.

**Predicting tumor cell line response to drug pairs with deep learning**

Fangfang Xia, Maulik Shukla, Thomas Brettin, Cristina Garcia-Cardona, Judith Cohn, Jonathan E. Allen, Sergei Maslov, Susan L. Holbeck, James H. Doroshow, Yvonne A. Evrard, Eric A. Stahlberg and Rick L. Stevens.

**High-throughput cancer hypothesis testing with an integrated PhysiCell-EMEWS workflow**

Jonathan Ozik, Nicholson Collier, Justin M. Wozniak, Charles Macal, Chase Cockrell, Samuel H. Friedman, Ahmadreza Ghaffarizadeh, Randy Heiland, Gary An and Paul Macklin.

**Scalable deep text comprehension for Cancer surveillance on high-performance computing**

John X. Qiu, Hong-Jun Yoon, Kshitij Srivastava, Thomas P. Watson, J. Blair Christian, Arvind Ramanathan, Xiao C. Wu, Paul A. Fearn and Georgia D. Tourassi.
